# Primary healthcare providers’ views on improving sexual and reproductive healthcare for adolescents in Bolivia, Ecuador, and Nicaragua

**DOI:** 10.3402/gha.v6i0.20444

**Published:** 2013-05-15

**Authors:** Lina Jaruseviciene, Miguel Orozco, Marcia Ibarra, Freddy Cordova Ossio, Bernardo Vega, Nancy Auquilla, Joel Medina, Anna C. Gorter, Peter Decat, Sara De Meyer, Marleen Temmerman, Alexander B. Edmonds, Leonas Valius, Jeffrey V. Lazarus

**Affiliations:** 1 Department of Family Medicine, Lithuanian University of Health Sciences (LUHS), Kaunas, Lithuania; 2 Centro de Investigación y Estudios de la Salud (CIES), Managua, Nicaragua; 3 South Group (SG), Cochabamba, Bolivia; 4 University of Cuenca (UC), Ecuador; 5 Instituto Centro Americano de Salud (ICAS), Managua, Nicaragua; 6 Internationation Centre for Reproductive Health (ICRH), Ghent University, Belgium; 7 Amsterdam University, Amsterdam, The Netherlands; 8 Copenhagen HIV Programme, Faculty of Health and Medical Sciences, Copenhagen University, Denmark

**Keywords:** adolescents, reproductive health services, primary healthcare, healthcare personnel, Latin America

## Abstract

**Objectives:**

To elicit the views of primary healthcare providers from Bolivia, Ecuador, and Nicaragua on how adolescent sexual and reproductive health (ASRH) care in their communities can be improved.

**Methods:**

Overall, 126 healthcare providers (46 from Bolivia, 39 from Ecuador, and 41 from Nicaragua) took part in this qualitative study. During a series of moderated discussions, they provided written opinions about the accessibility and appropriateness of ASRH services and suggestions for its improvement. The data were analyzed by employing a content analysis methodology.

**Results:**

Study participants emphasized managerial issues such as the prioritization of adolescents as a patient group and increased healthcare providers’ awareness about adolescent-friendly approaches. They noted that such an approach needs to be extended beyond primary healthcare centers. Schools, parents, and the community in general should be encouraged to integrate issues related to ASRH in the everyday life of adolescents and become ‘gate-openers’ to ASRH services. To ensure the success of such measures, action at the policy level would be required. For example, decision-makers could call for developing clinical guidelines for this population group and coordinate multisectoral efforts.

**Conclusions:**

To improve ASRH services within primary healthcare institutions in three Latin American countries, primary healthcare providers call for focusing on improving the youth-friendliness of health settings. To facilitate this, they suggested engaging with key stakeholders, such as parents, schools, and decision-makers at the policy level.

Since the International Conference on Population and Development was held in 1994, efforts to address adolescent sexual and reproductive health (ASRH) have intensified, but this remains an area of great concern. ASRH of adolescents in Latin America is of particular interest because of fertility patterns in the region. While adult women have experienced declining fertility since 1970, this is not the case for adolescent girls (aged 15–19): adolescent conception rates range from 12.7 to 20.5% in the region (1, 2). The increase in contraceptive use, including condoms, in Latin America has not proven sufficient to decrease the risk of adolescent pregnancy (3) nor other risks associated with sexually transmitted infections (STIs) (4). Limited data, underreporting, and the weakness of the surveillance systems impede the measurement of the magnitude of STI prevalence in the region (4). The estimated percentage of 15 to 24-year-old Latin American youths living with HIV is 0.2% in females and 0.7% in males (4). AIDS is the cause of 47% of all deaths by infectious and parasitic diseases for adolescents and youths aged 15–29 in the region (5).

The appropriateness and accessibility of ASRH services are widely recognized as factors that can contribute greatly to the improvement of ASRH (5). However, studies have documented shortcomings in how primary care centers respond to the ASRH needs (6). Many efforts have been made to identify barriers to appropriate and accessible ASRH services and to develop strategies for improving these services. Difficulties faced by adolescents with regard to ASRH care have been assessed extensively (7, 8). There are also considerable studies on the difficulties that providers encounter in trying to reach adolescents with sexual and reproductive health needs (9, 10). This has led to a wide-ranging set of recommendations to improve the existing services, such as providing a friendly reception to adolescent patients (11); ensuring that adolescent patients have time alone with physicians during consultations (12); advertising sexual health services in locations frequented by adolescents (13); and increasing adolescents’ awareness of opportunities to obtain services without the knowledge of their parents (14).

A study from Brazil revealed low awareness among adolescents of the existing health services and have also indicated that adolescents encounter administrative barriers and feel embarrassed when accessing such services (15). An emphasis on maternal care for adolescents in Ecuador is compounded by poor access to contraceptive services (16). In Jamaica, condoms are provided for less than 10% of sexually active adolescents and other birth control methods for 24% of sexually active adolescents (17). Prevailing biomedical approaches to primary healthcare generally and to sexual and reproductive healthcare specifically ignore psychosocial considerations (17–19).

Evidence indicates that in Latin America, the quality of SRH services for adolescents might be compromised by physicians’ limited expertise and experience related to ASRH (20). In addition, other provider-related barriers such as erroneous knowledge, outdated practices, unnecessary diagnostic tests and non-supportive attitudes can play an important role in adolescents not being able to access contraceptives (20). Researchers have also identified concerns about physicians sometimes having a paternalistic and gender-insensitive attitude towards adolescent patients (16) and about consultations being too brief (17).

The competence and attitudes of healthcare providers may affect health services and also the implementation of new policies and/or programs (21). However, there is little evidence regarding healthcare providers’ perspectives on how ASRH services can be improved.

In this study, based on qualitative data collected from physicians, nurses, other service providers, administrative staff, and medical students in Cochabamba (Bolivia), Cuenca (Ecuador), and Managua (Nicaragua), we explored the perceptions of how to improve ASRH services from the healthcare providers’ perspective. The selected cities have large teenage populations whose sexual and reproductive health situation is representative of urban adolescents in the region of the Andes (Cochabamba and Cuenca) and Central America (Managua). The prevention of unwanted adolescents’ pregnancies is a national priority in all three study countries (2, 22); however, the context of health service provision in selected areas differed. In Managua (Nicaragua), for example, the public health system provides the majority of health services and private health services are either unaffordable or unavailable in the neighborhoods covered by selected healthcare centers. The recently implemented Family and Community Health Model in Nicaragua did not envisage the creation of adolescents-only clinics (23, 24). In Cuenca and Cochabamba, however, private health services exist in parallel with the public health system and are commonly used by adolescents. The Andean plan to prevent adolescent pregnancies among other measures emphasizes the need for differentiated health services for adolescents in Bolivia and Ecuador (2).

## Methods

This article reports on one component of the Community Embedded Reproductive Health Care for Adolescents in Latin America (CERCA) study, a European Commission-funded interventional research project. CERCA seeks to contribute to global knowledge about how primary healthcare can be more responsive to the ASRH needs (24). Its immediate objective is to create a community-based model to improve ASRH in Bolivia, Ecuador, and Nicaragua. The CERCA study incorporates three methodological approaches: action research, community-based participatory research, and intervention mapping.

In the first phase of CERCA, determinants of the ASRH needs were assessed in three cities: Cochabamba, Bolivia; Cuenca, Ecuador; and Managua, Nicaragua. The findings of the assessment will guide the development of a comprehensive strategy for improving access to adolescent-friendly reproductive health services at primary healthcare facilities; creating an enabling environment; and improving adolescents’ reproductive health decision-making skills.

Qualitative data were collected from physicians, nurses, other service providers, administrative staff, and medical students. Personnel from seven primary health centers (two in Bolivia, three in Ecuador, and two in Nicaragua) that are involved in the CERCA project were invited to take part in the study. This study was the introductory component of future interventions that aimed to involve all health center personnel, taking into account that the transfer of knowledge to colleagues is rather low (25). Thus, all personnel, including auxiliary administrative staff, and medical students who were in training at health centers during the study, were invited to take part in the study. Discussion meetings were scheduled in all of the seven centers; in two centers two discussions were performed. Everyone who agreed to participate in the study provided written informed consent. Participants were assured confidentiality and were informed as to how the data collected from them would be used.

A focus group discussion method could not be used in our study due to the greatly varying number of participants in each group of discussions, the non-homogeneity of the professional background of participants and their uneven status in the health setting's hierarchy. However, facilitated discussions of all health personnel regarding the health center's possibility to improve ASRH care were considered an important component of the project itself. Therefore, discussions were held but only the written answers to the questions are analyzed in this article. Researchers conducted nine facilitated group discussions with study participants. The number of personnel in each varied and the number of potential participants was not known prior to the start of the meetings. Groups ranged in size from 6 to 24 study participants. The total number of participants in this convenience sample was 126. The number of participants in each group was as follows: 24, 10, and 12 in Bolivia; 17, 6, and 16 in Ecuador; and 17, 15, and 9 in Nicaragua. Each group consisted of personnel from just one health center. Prior to each discussion, study participants were asked to provide written answers to the following questions:

- What are the reasons why adolescents do not usually go to health centers for sexual and reproductive health services?
- What difficulties do health center personnel encounter in providing ASRH services?
- What measures could be implemented in health centers to improve ASRH care?

The answers were collected and summarized during the period when participants were introduced to the CERCA project objectives. Participants’ insights served as prompts for future discussions that lasted approximately 90 min each. The main focus of discussions was to set the main directions for interventions that could be applied in particular health centers during the duration of the CERCA project. Two CERCA staff attended each discussion, one acting as a moderator and the other taking notes. The discussions were not recorded.

Only written responses produced by the participants to each specific question are analyzed in this article. The responses of each participant were transcribed, translated from Spanish to English, and subsequently analyzed. Transcripts were analyzed employing content analysis methodology (26). Initial codes were developed after a careful reading of the transcripts. In the next stage of analysis, the codes were clustered into emergent categories. These categories were structured and grouped to determine the final themes. To ensure that the categories and themes consistently represented the data from all participating countries, final verification checks were made for each country data set. Only the theme-supporting policies to improve ASRH had categories that were not represented with data from all countries. The themes were then reviewed and refined prior to the development of the following summary of results. As participants’ written responses were anonymous, in the text we provide information about the country and the number of discussion in the country.

This study was approved by the Bioethics Committee of Ghent University, Belgium, in 2011. The management of the participating health centers of Cochabamba, Cuenca, and Managua gave permission for the study.

## Results

**Chart 1:**
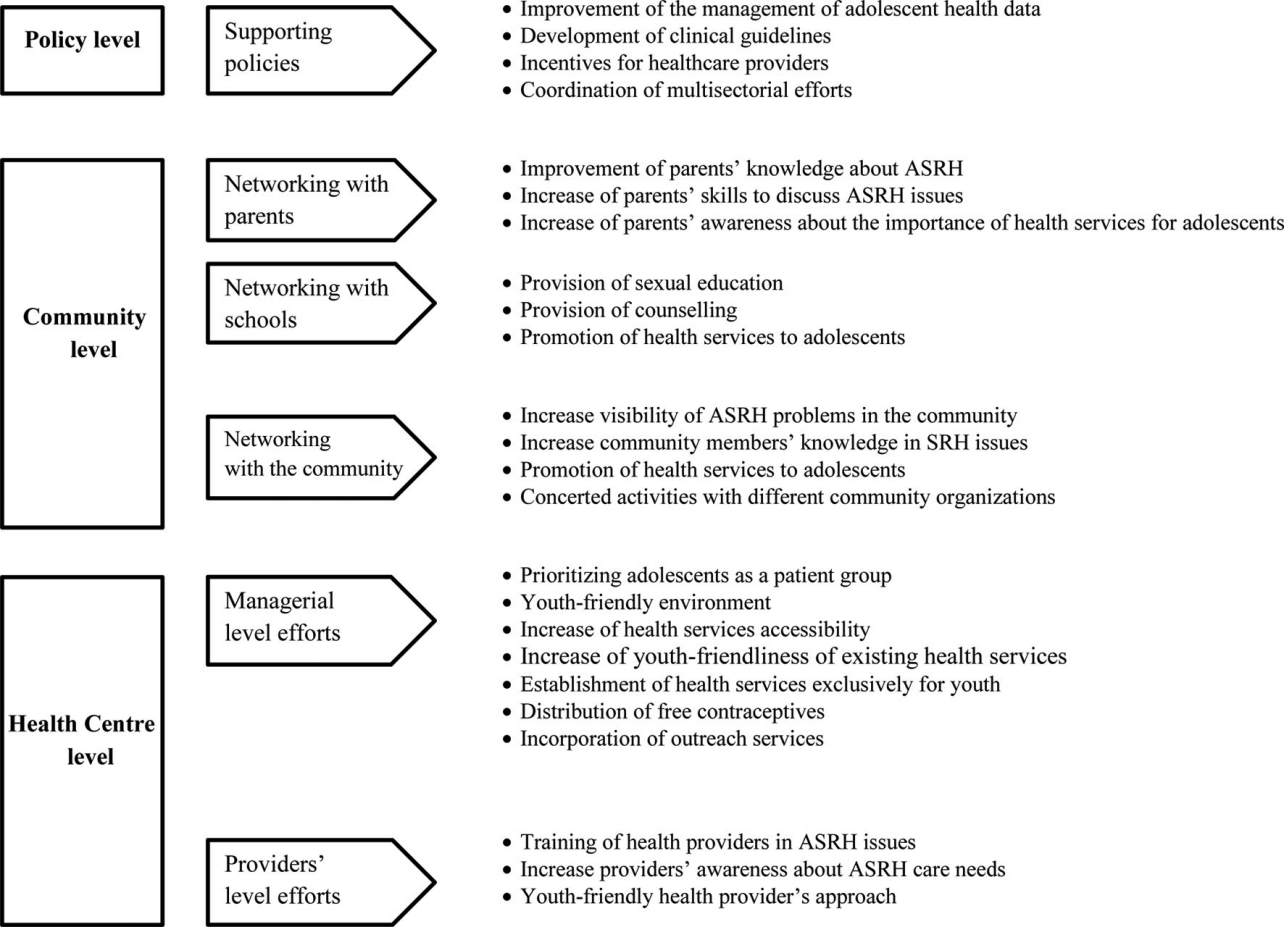
Potential to improve ASRH according to healthcare providers.

Demographic information about study participants is presented in “Study Participants” section, and qualitative findings are presented in four additional subsections. There were four final themes defined: managerial-level efforts to improve ASRH services: health provider-level efforts to improve ASRH; networking with schools, parents and the community; and supportive policies to improve ASRH. These themes reflect the potential for ASRH improvement at healthcare centers, and at community and policy levels. The categories that emerged from the analysis and eventually formed the final themes are presented in Chart 1.

### Study participants

The study enrolled 126 people working at primary healthcare facilities: 46 from Bolivia, 39 from Ecuador, and 41 from Nicaragua (Table 1). Male study participants outnumbered female study participants. In Bolivia and Ecuador, healthcare providers of all age groups participated in the discussion groups. In Nicaragua, there was an underrepresentation of the age groups younger than 31 and older than 50 years. Almost half of the participants were physicians, and approximately one-third was nurses. There was less representation of psychologists, educators, medical students, and administrative staff. Medical students took part in the discussions, in only two healthcare centers of Bolivia; in other centers, there were no medical students at the time the study was performed.

**Table 1:** Demographic breakdown of study participants

Indicator	Bolivia	Ecuador	Nicaragua	Total
Gender				
Male	15	10	8	33
Female	31	29	33	93
				
Age				
30 years and younger	14	10	5	29
31–40	13	11	19	43
41–50	12	7	15	34
51 and older	7	11	2	20
				
Occupation				
Physician	14	19	25	58
Nurse	16	11	15	42
Psychologist, educator	6	2	0	8
Medical students	7	7	1	7
Administrative staff	3			11
Total	46	39	41	126

### Managerial-level efforts to improve ASRH services

Study participants in all three countries put the greatest emphasis on managerial-level interventions to improve ASRH services. Prioritizing adolescents as a patient group and being attentive to their sexual health problems were the most common suggestions for making health institutions more adolescent-friendly. Next, the physical environment was addressed. A participant from Nicaragua said, ‘It's necessary to have a more attractive environment to attract adolescents’ (Nicaragua 2). Participants also believed that adolescents’ access to healthcare could be increased by eliminating formalities. One said, for example, ‘Don't refuse a family planning consultation just because they have missed an appointment’ (Nicaragua 3). Another called for ‘specific [hours of service] for adolescents taking into account the school hours’ (Bolivia 1).

Study participants also called for the development of new services such as the distribution of free contraceptives. A participant from Ecuador proposed ‘medical personnel visits to the communities [to reach] young people who work and don't go to school, and who don't have knowledge about contraceptives’ (Ecuador 1). Some providers endorsed the creation of separate programs specifically targeting adolescents. A participant from Ecuador was in favor of ‘establishing health centers exclusively for adolescents’ (Ecuador 3), and a Nicaraguan participant recommended having ‘physicians and nurses specifically working for adolescents’ (Nicaragua 2). Conversely, other study participants stated that a comprehensive multidisciplinary approach could be instrumental in addressing ASRH care needs without creating vertical programs: ‘when the opportunity arises; each professional should... meet with the adolescent and discuss relevant sexual health issues’ (Bolivia 2).

### Health provider-level efforts to improve ASRH services

Primarily, the need for a more friendly approach with adolescents by health providers was clearly voiced. It was suggested that increasing awareness among providers for ASRH care needs would be a major step in improving service delivery. Participants also thought that it was important to make providers more aware of their responsibility to deliver the best healthcare possible even with limited resources. Different aspects of an adolescent-friendly approach were mentioned. For example, a participant from Nicaragua brought up that providers should have good counseling skills and the ability ‘to talk with adolescents in an amiable way’ (Nicaragua 1). A participant from Ecuador said, ‘Don't talk to patients using words that they don't understand’ (Ecuador 1). According to another Ecuadorian participant, there was also a need ‘to treat adolescents with more respect from the moment they make an appointment until they leave the health center’ (Ecuador 2). Participants additionally stressed the primacy of maintaining confidentiality and privacy for adolescent patients: ‘give more privacy to adolescent during consultations, especially if he wants to be listened only by physicians and not by students’ (Bolivia 1).

Additional training for all personnel at health centers was an almost unanimous suggestion by study participants. It was believed that training would improve providers’ knowledge of ASRH care and make them more aware of relevant guidelines and programs. Training was also seen as a way to develop skills for communicating with adolescents and comprehensively assessing their health problems.

Other participants were in favor of involving psychologists in the provision of ASRH services. A Bolivian study participant said that there is a need ‘to have good and accessible psychologists to provide quality care for patients’ (Bolivia 3).

### Networking with schools, parents, and the community

Study participants identified collaboration with schools as the most important outreach activity to improve ASRH care at primary health centers. Participants considered primary healthcare providers to be partners of schools with regard to the provision of sexuality education. They suggested that this partnership should extend beyond formal classes and traditional teaching activities in schools by including peer education programs, social networks and new media, such as interactive websites, video games. A participant from Ecuador commented on a ‘massive campaign about sexual and reproductive issues in [internet] social networks that are highly used at the moment by adolescents’ (Ecuador 3). A participant from Bolivia proposed establishing ‘free-of-charge telephone lines to improve adolescents’ access to sexual information’ (Bolivia 1). Participants furthermore suggested using schools as settings for medical counseling on ASRH issues ‘once per week physicians could go to school to give the consultations on the sexual and reproductive health issues for everybody who needs that’ (Ecuador 1). Finally, they perceived schools as promoters of healthcare services, with one participant from Ecuador saying, ‘Education in sexual health and the promotion of medical services is essential at all schools in the area’ (Ecuador 2).

Study participants also viewed parents as potentially significant partners for improving ASRH care. Study participants suggested that parents themselves need sexuality education in order to address sexual matters more skillfully with their children: ‘to train parents and teachers in this topic to enable them to guide an adolescent’ (Bolivia 3). A Nicaraguan study participant wanted to see efforts to ‘involve the parents in activities’ (Nicaragua 3) and an Ecuadorian study participant thought that such efforts could be instrumental ‘for increasing parents’ awareness about the importance of allowing their children to attend health centers (Ecuador 2).

Many participants wanted to see broader community involvement in the response to ASRH needs as well. A participant from Ecuador talked about making the problems facing adolescents ‘visible in the community’ (Ecuador 1). There were also suggestions to involve community leaders and community organizations in activities addressing ASRH.

According to study participants, the interaction between health centers and communities in relation to ASRH should have two main purposes. The first is to improve community members’ knowledge of ASRH needs. A Nicaraguan participant talked about the need ‘to provide the information to the adults to break with myths and taboos’ (Nicaragua 2). The second purpose is to promote ASRH services and different health promotion activities provided by health centers. Community involvement in health center activities is seen as essential to ‘ensure an environment that is supportive of improving adolescents’ knowledge on sexual and reproductive health’ (Bolivia 2).

### Supportive policies to improve ASRH

To a lesser extent, participants suggested how policy measures could contribute to better ASRH care: by promoting the development of clinical guidelines; by improving the management of adolescents’ health data; by creating incentives for healthcare providers; and by coordinating a multisectoral approach. The development of policies should be accompanied by increased financing – ‘the project calls for young people's involvement, but at the same time the authorities do not give adequate financing’ (Nicaragua 1).

## Discussion

This qualitative study explored primary healthcare providers’ views on how ASRH services in Bolivia, Ecuador, and Nicaragua can be improved. Physicians, nurses, and other personnel at seven primary healthcare clinics identified a broad array of concerns at health center, community, and policy levels that could contribute to improving ASRH care.

Study participants put the greatest emphasis on managerial-level interventions such as prioritizing adolescents as a patient group. They also stressed the importance of healthcare provider-level efforts such as training activities to improve providers’ skills to interact appropriately with adolescents. Study participants made suggestions for outreach activities particularly for schools. They additionally named parents and the broader community as partners to be engaged in efforts promoting sexual and reproductive health services for adolescents. Study participants’ suggestions for improving ASRH services within primary healthcare institutions mainly concerned making the facilities more youth-friendly. The strategies suggested by study participants to achieve this objective composed of both managers and service providers. Other studies had indicated the efficiency of multifaceted initiatives emphasizing the importance of the managers’ participation in implementing innovations (21, 27), as well as highlighting the value of regular orientation and training for health providers (25).

Concordantly with the providers’ perspective in our study, research shows that the development of health facility policies and establishing internal standards for the provision of ASRH could be instrumental in coordinating efforts at the managerial level and provider level (28). Our findings indicate that the elaboration of procedures concerning ASRH services in particular may encompass issues, such as registration and confidentiality. Furthermore, written office policies seem to improve provider's knowledge of the existing guidelines and to increase the probability of adherence to these guidelines (9).

Study participants’ proposed strategies for engaging schools, parents, and the community in adolescent sexual and reproductive healthcare have the potential to both promote the existing services and create a supportive environment for adolescents to seek out those services. Schools, parents, and the community in general are well placed to integrate issues related to sexual and reproductive health in the everyday life of adolescents. By reducing the taboos on sexuality, schools, community organizations, and adolescent parents could become ‘gate-openers’ for the existing ASRH services. However, families are not always ‘safe-spaces’ for adolescents (29) and adults are not always knowledgeable about the existing local services (30). This is why awareness raising on ASRH issues among parents and community should be complemented by educational components. Other studies have identified educational efforts aimed at parents and the community as effective instruments for allying parents with health providers (31); improving adults’ knowledge of local contraceptive services and changing their negative attitudes regarding the sexual behavior of young people (30); highlighting the benefits of sexual and reproductive care; and destigmatizing health services and procedures (32). Study participants’ suggestions about using technology to enhance ASRH education and care call attention to a promising approach that has been welcomed by young people (33). Research demonstrates that Internet-based delivery of the components of the interventions has a little, but statistically significant effect on health-related behavior (34).

Healthcare providers who took part in the study emphasized the need to influence the cultural aspects that interfere with adolescents’ access to sexual and reproductive health services. However, study participants did not apparently perceive the need to change their own personal perspectives and norms towards ASRH issues that could affect adolescent consultations on sexual and reproductive health problems. Several studies demonstrated the effect of the social context on the attitudes and of health professionals. A qualitative study among providers in the Amazon basin of Ecuador concluded that moralistic attitudes and sexism among providers were limiting services’ capability to promote girls’ sexual and reproductive health services and rights (29). Similarly, a qualitative study among midwives and physicians demonstrated how the cultural and societal context of the healthcare providers determined their attitude and practice (35). Thus, the introspective reflection of healthcare providers on how their own sexual norms and perspectives on adolescents could influence their practice and performance related to the provision of ASRH could be a valuable component of educational interventions to healthcare providers. The training should also cover issues such as gender-based violence, gender inequality, and abortion, which were not addressed by study participants.

### Limitations of the study

This study had several limitations. First, the groups of study participants were not similar across the countries: medical students were present only in Bolivia. In Nicaragua, groups consisted nearly exclusively of healthcare providers: physicians and nurses. Physicians dominated in Nicaragua, nurses, auxiliary medical staff, and medical students in Bolivia. However, the categories and themes, except the last theme (supporting policies) consistently represented the data from all three countries, indicating similarities in the perspective of health center personnel. The views and experiences of the participants may not represent those of the larger primary healthcare providers’ community since the study included only providers from those primary healthcare facilities that took part in the CERCA project. As all of the study population was based at urban clinics, their perspective may be different from those of staff at rural clinics. Moreover, the study explored healthcare provider's perspectives on ASRH, but their perspectives may differ from community's perspectives and adolescents’ perspectives. Healthcare providers, especially physicians, who constituted almost half of our study participants, often have a higher socio-economic status than the people they treat, and this may affect how they think about healthcare issues. However, this study did not intend to provide a comprehensive assessment of the situation.

Second, some of the questions posed, might have led study participants to focus on health center initiatives to improve ASRH care. The findings revealed that most health providers’ insights were related to health center and community levels. Very few suggestions addressed the policy level.

The third possible study limitation is related to the method of the data collection. As study participants provided their written insights on discussed issues, some richness and depth of the data that could be expected from participants interacted in groups were lost (36).

Although it is possible that verbal interaction in the group of primary healthcare providers would have helped to reveal more aspects related to the topic, we believe that the chosen approach gave voice to all participants regardless of their status in the health setting's hierarchy (people with less power and authority in the healthcare center e.g. nurses and health auxiliaries were in discussion alongside people with more power and authority, e.g. doctors and center managers) and elicited a diverse range of opinions. On the other hand, we believe that participation of all personnel in the development of interventional strategies favorably affected the selection of the most appropriate initiatives for each center and increased the commitment of healthcare providers to project activities that were in line with the idea of community-based participatory research.

The individuals collecting the data (moderator and note-taker) were CERCA staff members. This may have influenced study participants’ opinions. However, as participants identified the factors in written form before the discussions were held, the influence of CERCA staff was likely to be limited.

## Conclusions

Our survey results revealed that study participants are aware of the barriers faced by adolescents in sexual and reproductive healthcare. Their suggestions on the improvement of ASRH care addressed the vast majority of barriers that Jacobs et al. (27) identified as key targets for the interventions to improve health services accessibility. However, the study findings suggest that the implementation of these interventions could not be left solely to health providers; a collaborative approach involving managerial and community levels is needed.

Future research should expand on the findings of this study by using quantitative methods and by looking at the broader context. For example, studies could consider the perspectives of adolescents and other community actors. Furthermore, a wider reassessment of ASRH care in the cities of Cochabamba (Bolivia), Cuenca (Ecuador), and Managua (Nicaragua) should be undertaken.

This study's results could inform the design of strategies to engage healthcare providers in the improvement of ASRH services. Realistic evaluation of the implementation of youth-friendly health services in Ecuador demonstrated that a successful transformation process from ‘ordinary’ health facilities to youth-friendly health services was triggered by an interaction between the healthcare team and the community agencies that supported innovation rather than by healthcare team itself (37).
